# 3D-glass molds for facile production of complex droplet microfluidic chips

**DOI:** 10.1063/1.5013325

**Published:** 2018-04-03

**Authors:** Miguel Tovar, Thomas Weber, Sundar Hengoju, Andrea Lovera, Anne-Sophie Munser, Oksana Shvydkiv, Martin Roth

**Affiliations:** 1 Leibniz Institute for Natural Product Research and Infection Biology, Hans Knoell Institute, Beutenbergstr. 11a, 07745 Jena, Germany; 2 Friedrich Schiller University, Fuerstengraben 1, 07743 Jena, Germany; 3 Ilmenau University of Technology, Ehrenbergstr. 29, 98693 Ilmenau, Germany; 4 FEMTOprint SA, Via Industria 3, 6933 Muzzano, Switzerland; 5 Fraunhofer Institute for Applied Optics and Precision Engineering-IOF, Albert-Einstein-Str. 7, 07745 Jena, Germany

## Abstract

In order to leverage the immense potential of droplet microfluidics, it is necessary to simplify the process of chip design and fabrication. While polydimethylsiloxane (PDMS) replica molding has greatly revolutionized the chip-production process, its dependence on 2D-limited photolithography has restricted the design possibilities, as well as further dissemination of microfluidics to non-specialized labs. To break free from these restrictions while keeping fabrication straighforward, we introduce an approach to produce complex multi-height (3D) droplet microfluidic glass molds and subsequent chip production by PDMS replica molding. The glass molds are fabricated with sub-micrometric resolution using femtosecond laser machining technology, which allows directly realizing designs with multiple levels or even continuously changing heights. The presented technique significantly expands the experimental capabilities of the droplet microfluidic chip. It allows direct fabrication of multilevel structures such as droplet traps for prolonged observation and optical fiber integration for fluorescence detection. Furthermore, the fabrication of novel structures based on sloped channels (ramps) enables improved droplet reinjection and picoinjection or even a multi-parallelized drop generator based on gradients of confinement. The fabrication of these and other 3D-features is currently only available at such resolution by the presented strategy. Together with the simplicity of PDMS replica molding, this provides an accessible solution for both specialized and non-specialized labs to customize microfluidic experimentation and expand their possibilities.

## I. INTRODUCTION

Microfluidic experimentation is quickly revolutionizing various scientific fields with explicit rapid progress in chemical and biotechnological research.^1–6^ The microfluidic chip (structures with channels in the micrometric scale) is the heart of any microfluidic approach, in which key functionalities define the advantages of its application. While multiple vendors already offer predetermined chips at relatively convenient prices, adapting its design for a certain application will in most cases require further customization and multiple rounds of iterative optimization. Therefore, custom-made production is the most common bottleneck for the adoption of microfluidic strategies, particularly by non-microfluidic labs.^7,8^

In the late 1990s, Whitesides *et al.* introduced the fabrication of microfluidic structures using a combination of photolithography and soft lithography,^9^ which triggered the rise of microfluidic research due to its relatively easy implementation. In fact, most laboratories are able to perform polydimethylsiloxane (PDMS)-based soft lithography, but the major constraint is the production of molds utilizing photolithography. This is because photolithography—originally developed for microelectronic fabrication—requires special steps and equipment such as access to a clean room, as well as surface coating, micro-alignment, and light exposure devices.^10^ Moreover, photolithography imposes an additional limitation in the construction of microfluidic structures since they must be designed as a series of two-dimensional structures within one chip, and thus each channel will have a constant depth or height. This has strongly restricted the possibilities (and creativity) of microfluidic engineers for designing useful microchannel structures. Nevertheless, reports of novel functions and improved handling have been achieved with designs that require multiple depth levels, confinement gradients, semicircular channels, drip molds, micro-textured surfaces or structures with high aspect ratios, highlighting the need for more complex three dimensional structures.^11–14^

The rapid evolution and dissemination of 3D-printing technologies is certainly impacting the design and fabrication of microfluidic devices^15^ with potential to solve all the aforementioned constraints. Additive manufacturing techniques have gained hype with the possibilities they represent at large scales, and therefore have been intensively developed to work at the microscale. However, most current applications are strongly restricted due to limitations in the resolution, surface roughness, and material selection.^16,17^ Another 3D-microfabrication strategy, which is not based on additive manufacturing, is the use of femtosecond lasers irradiation followed by chemical etching.^18–21^ Like additive manufacturing techniques, it does not require clean-room facilities, can be automated, and provides flexibility to obtain 3D structures without significant increase in fabrication complexity and time.^22^ Yet, it offers the crucial advantages of easily achieving sub-micrometric resolution and that can be applied in different materials,^23^ but most prominently in glass. Therefore, this strategy has been thoroughly used in recent years for the production of complex microfluidic structures,^24,25^ some including optical and electronic elements.^26,27^

Yet, despite the possible fabrication advantages, the equipment and expertise required by these strategies is still a bottleneck for widespread usage and cannot yet replace PDMS replica molding in terms of simplicity and cost-effectiveness. A comparatively simple and effective solution is then to use the advantages of 3D-prototyping to create molds that can be subsequently casted in PDMS.^28,29^ This enables the production of structures with sub-micrometer resolution, high aspect ratios, and the straightforward production of pseudo-3D-features. This could prove specially relevant in the particular case of droplet microfluidics, where the rapidly growing number of non-microfluidic adopters require the flexibility to easily fabricate chips that fulfill innovative applications.

This paper presents complex three-dimensional PDMS chips for droplet microfluidics made from 3D-glass molds produced using the femtoprint process.^30^ We demonstrate the applications of high resolution molds in channels with changing depth, stable picoinjection, detection with optical fibers, and ultra-fast droplet generation using confinement gradients.

## II. RESULTS AND DISCUSSION

### A. Mold fabrication

**FIG. 1.**
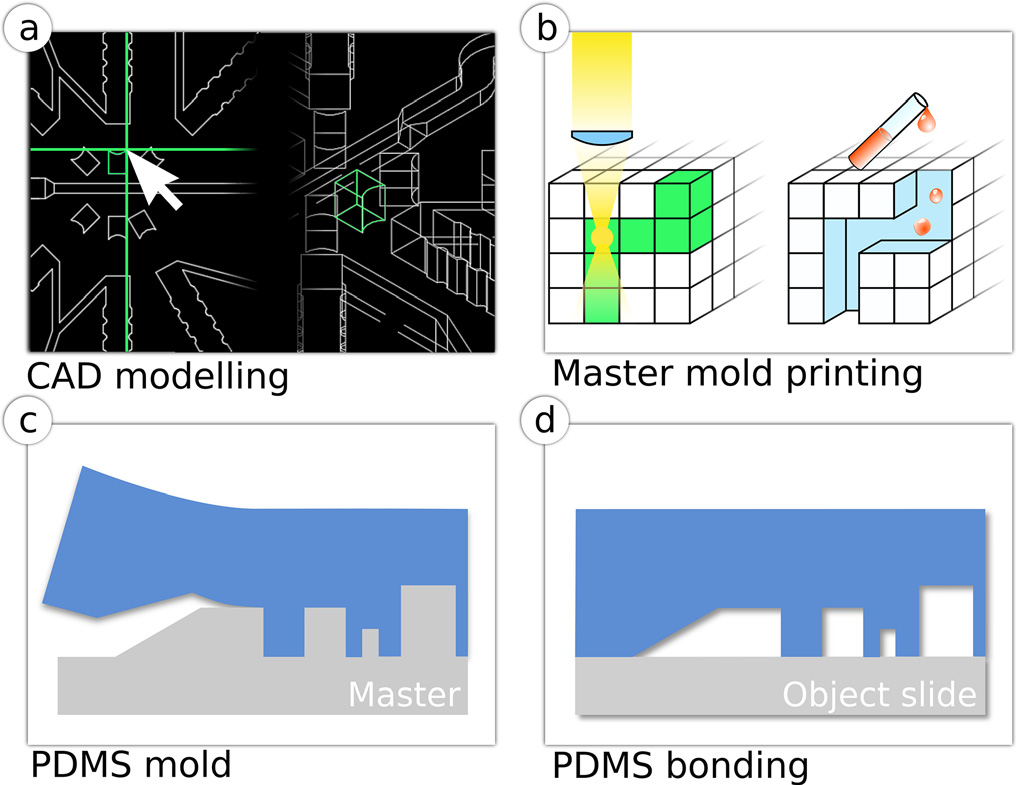
Production of a PDMS chip from 3D-glass molds. (a) Construction of the three-dimensional CAD model. (b) Fabrication of a glass master mold with femtosecond laser machining. (c) Soft-lithographic production of a PDMS mold (blue) from the glass master mold. (d) Plasma bonding of PDMS stamp to an object slide.

Using the 3D-CAD design of the chip structure [Fig. 1(a)], a femtosecond laser was used to replicate the design within the fused silica substrate as a service by FEMTOprint SA. When the laser is focused within the material, the local properties in the focal volume are tuned with sub-micrometric resolution achieved by a non-linear absorption process. Thus, the etching rate of the substrate is increased up to 200 times with respect to the pristine material,^31^ enabling precise removal of the exposed substructures and creating the 3D-mold [Fig. 1(b)]. The fabrication time can be from minutes up to hours depending on the design size, with writing speeds that can be up to 40 mm/s and laser repetition rates of up to 1 MHz. Depending on the design strategy, either positive or negative molds can be manufactured. In case of a negative mold, an intermediate PDMS mold is generated from the glass mold, from which final PDMS chips are subsequently casted [Figs. 1(c) and 1(d)].

The 3D-glass molds and PDMS chips were then characterized with a 3D-optical interferometer (Contour GT-K, Bruker) or a white-light interferometer (Zygo) to check the widths and depths of the channels and the local roughness on various locations. In the glass molds and the PDMS, the channel widths were made with ±1 *μ*m tolerance while the Z-axis divergence averaged ±3 *μ*m (Table S1, supplementary material). White-light interferometry images and data show the progressively changing depth of the structures in both the mold and the PDMS chips (Fig. S1, supplementary material). Typical Ra (average roughness) values for the machined parts are of 80 nm on the vertical sidewalls and of 200–300 nm on horizontal and 3D-structures (e.g., ramps). While this is ten times higher roughness than what is observed on PDMS structures fabricated from SU-8 molds (10–30 nm), we have not observed any significant alterations in fluidic or optical performance for our applications. Nevertheless, it is possible to additionally post-treat the glass molds to reduce roughness to similar values as in SU-8 (Fig. S2, supplementary material).

To demonstrate the functionality of this fabrication strategy, we have focused on chip designs for droplet microfluidic applications. This is an extremely fast-growing branch with a clear outreach towards non-microfluidic labs, as is the case in our microbiological research. We designed structures that provide tailor-made improvements in common droplet operations (generation, observation, picoinjection, and fiber-optic detection) but are too complicated or unsuitable to be produced with traditional photolithography.

### B. Multi-level structures for droplet trapping and fiber insertion

**FIG. 2.**
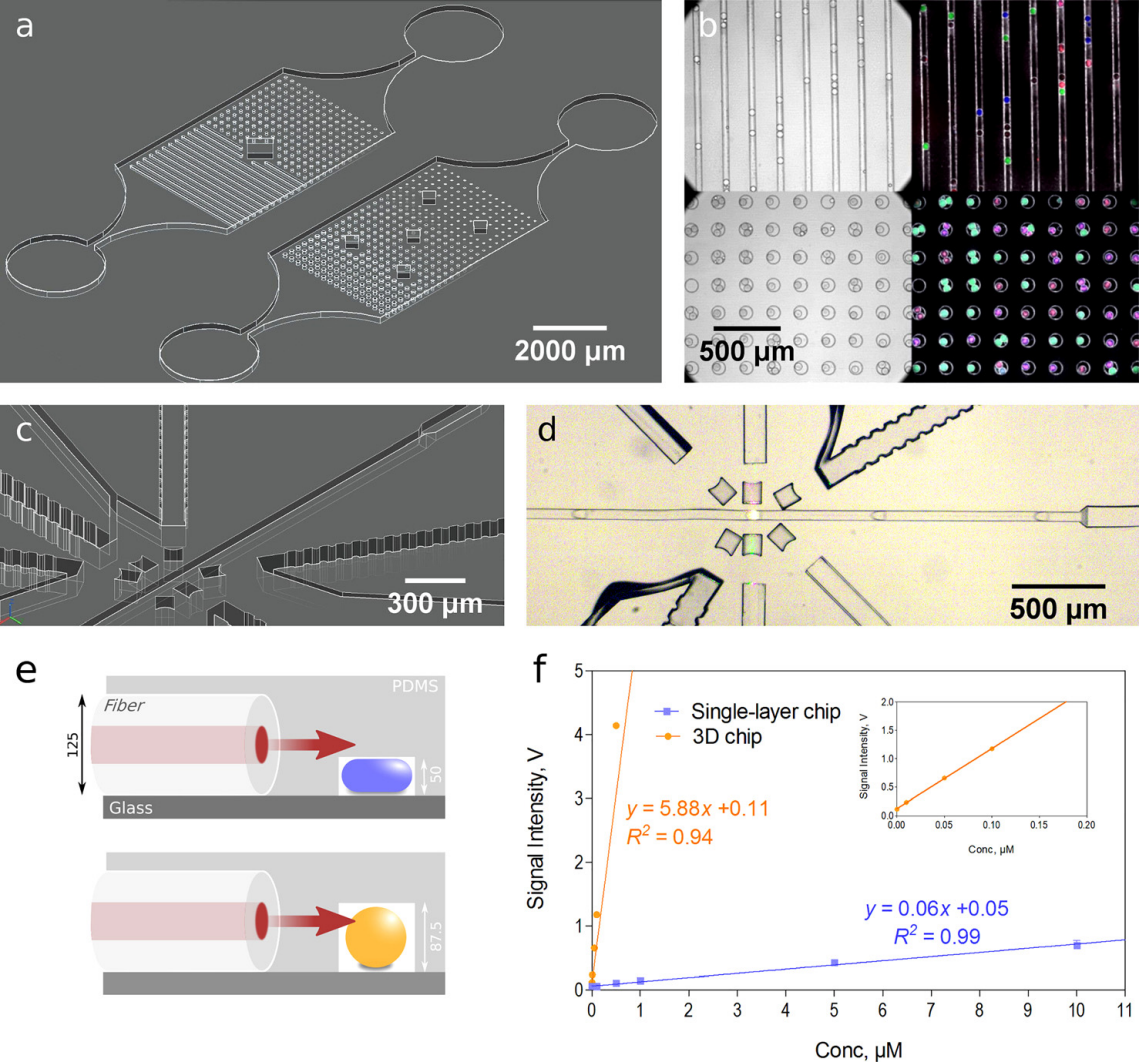
Droplet trapping chip for prolonged observation and imaging, including differently shaped chambers of variable depth (a). The incubated and imaged droplets encapsulate growing microorganisms (*Streptomyces* sp. and red fluorescent *E. coli*) labeled with distinct fluorescent markers (b). Multilevel structure for the fabrication of chips with facile optical fiber insertion and optimal droplet detection at varying channel heights [(c) and (d), (e)]. Calibration curves for detected light comparing a single-layer channel of 50 *μ*m height with the presented multilevel structure. Each data point represents the average for more than 1000 droplets per condition; the error bars represent one standard deviation, with the inset detailing lower concentrations (f). Multimedia view: https://doi.org/10.1063/1.5013325.1
10.1063/1.5013325.1

As a first application, we developed molds for chips with rail and anchor structures, allowing trapping of droplets^32^ and thereby their prolonged observation, which is crucial in many microbiological applications. The usage of different heights within one chip is key to design and select functional droplet traps. Classical manufacturing of such chips is extremely cumbersome due to the requirement of multiple photolithography steps with precise alignment, unmanageable in a non-microfluidic lab without mask aligners. Contrarily, the production of observation chips from 3D-glass molds is straightforward, resulting in structures ideal for droplet trapping and prolonged observation of growing microorganisms [Figs. 2(a) and 2(b); Fig. S3, supplementary material]. Multiple structure geometries and depths can be tested within one chip design, enabling flexibility for different applications or rapid testing for defining the ideal dimensions. Having chips with different channel depths is also essential for the insertion of optical fibers, which can be used for direct optical measurements in a simplified setup.^33,34^ The possibility of having multiple layers in the 3D printed mold allows easy chip fabrication with different depths for fluidic and fiber channels [Figs. 2(c) and 2(d) (Multimedia view); Fig. S4, supplementary material]. With our multi-level chips, light from the excitation fiber is properly guided to the fluidic channel, such that the light path completely reaches the flowing droplets. Whereas in a single layer chip, only a portion of light gets through the fluidic channel, limiting the excitation of the droplet contents and the collection of emitted light. In an experiment with fixed laser power and photomultiplier tube (PMT) gain, a signal increase in 100 times is measured at the same dye concentrations in our chip compared to the single-layer chip. Thus, the detection limit and dynamic range are also improved [Figs. 2(e) and 2(f)]. Furthermore, a deeper fiber channel allows easy and quick insertion of optical fibers into the chip (as fiber channels have the same depth as the diameter of the fiber).

### C. Ramps for improved flow manipulation

**FIG. 3.**
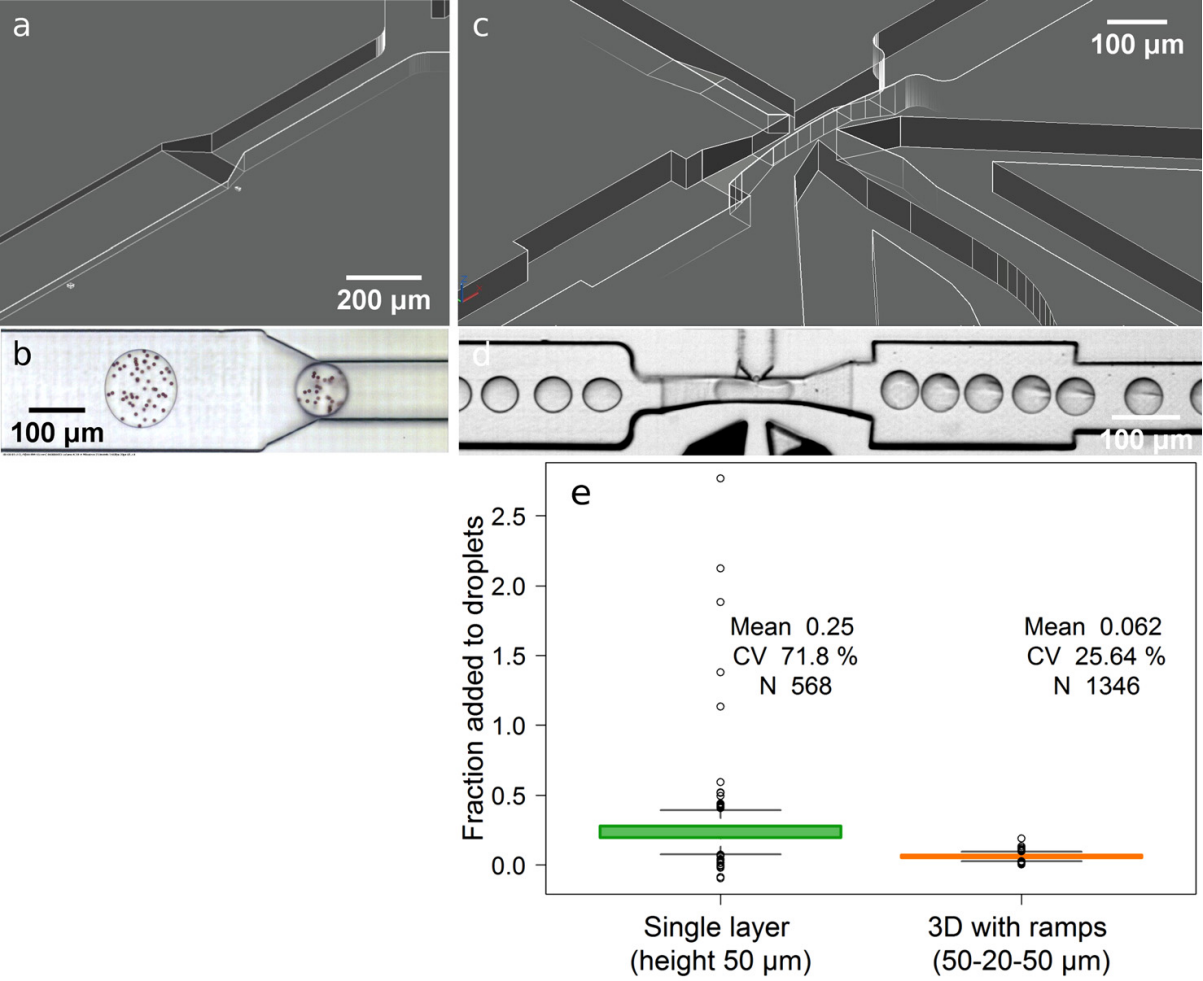
Design approaches with ramps to observe droplet contents in flow under different channel heights shown as sketch (a) and micrograph [(b)]; as well as to improve picoinjection [(c) and (d)]. Variability of the picoinjected fraction to droplets with a single layer structure compared to a multileveled structure with ramps (e). Multimedia views: https://doi.org/10.1063/1.5013325.2
10.1063/1.5013325.2
; https://doi.org/10.1063/1.5013325.3
10.1063/1.5013325.3

Different channel heights are not only useful for better microscopic observation of droplets and their contents, but also for flow manipulation and control. However, sudden increases or decreases in channel height can easily result in flow disturbances [droplets do not enter the restricted channel unless strongly pushed (supplementary material Video 1)] or even breakage of flowing droplets [as in step emulsification^35^ (supplementary material Video 2)]. A simple and intuitive solution would be to use structures with a gradual change in the channel height, like ramps. Yet, their usage has rarely been reported as a microfluidic strategy, since most accessible microfabrication tools have limited production abilities. However, ramps can be easily produced with the presented strategy [Figs. 3(a) and 3(b) (multimedia view); Fig. S5, supplementary material]. The use of ramps enabled stable droplet transition between different channel heights during generation and reinjection; which is critical for accurate image analysis of droplet contents.^36,37^ They also facilitate stable picoinjection [Fig. 3(d) (multimedia view); Fig. S6, supplementary material] as the flow stability of the picoinjected liquid and reinjected droplets increased with more constricted channels at the picoinjection nozzle, which is especially useful when handling relatively large droplets (>100 pl). While the picoinjected volumes are lower, it results in reduced variability of the picoinjected volume [Fig. 3(e)].

### D. Parallelized droplet generation

**FIG. 4.**
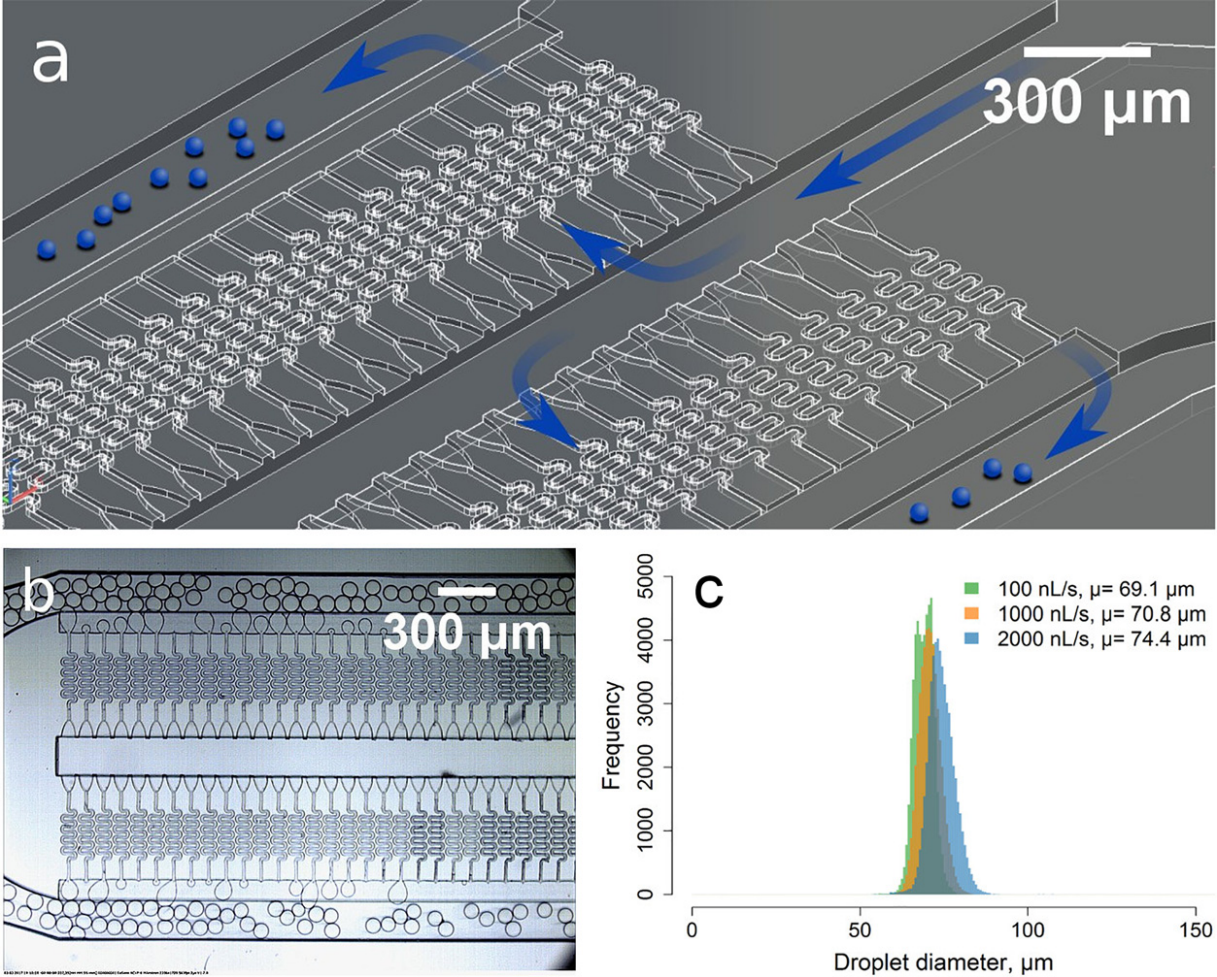
Multi droplet generator with 192 nozzles generating ≥5000 monodisperse droplets per second [(a) and (b)]. Histograms of droplet sizes produced at different flow rates, from 50 000 analyzed droplets (c). Multimedia view: https://doi.org/10.1063/1.5013325.4
10.1063/1.5013325.4

A combination of multiple levels and ramps was exploited with the development of a 3D-mold for the production of chips that enable ultra-fast droplet generation using gradients of confinement.^38^ As the required gradual and/or large changes in channel depth can be easily and more precisely manufactured with this approach, we were able to create a structure with 192 nozzles packed in a design of 25 mm length and 4 mm width including all inlets and outlets [Fig. 4(b) (multimedia view); Fig. S7, supplementary material] generating monodisperse droplets of ∅70 *μ*m (CV 4%.) with minimal dependency on the flow rates [Fig. 4(c), supplementary material Videos 3, 4], which can be higher than 5 *μ*l/s. The structure can be easily scaled to contain 1000 nozzles in a 6.5 cm structure. To the best of our knowledge, there is no other straightforward methodology that would enable the production of such architecture with structures ranging from 15 *μ*m to hundreds of micrometers in all dimensions and continuous changes in depth.

For the microfluidic chip designer and user, the presented mold/chip fabrication approach also improves a few practical features. The possibility to create 3D-structures provides higher complexity and therefore needs more time for design. However, the fabrication process is simpler and faster, requiring less time and expertise (mask-less and out-of-clean room method, fast prototyping). The fused silica mold is very stable, suffering no damage after more than ten rounds of molding. While the cost of a FEMTOprint 3D-glass mold slightly depends on the design complexity, it ranges between 1000 to 3000 EUR, which is generally only marginally more expensive than the fabrication of double layered SU-8 molds on a silica wafer when outsourced, which ranges between 1500 and 2000 EUR. More layers definitively increase the price and yet are more prone to fabrication errors.

## III. METHODS

### A. CAD design

Fluidic channel structures were initially designed in 2D using AutoCAD^®^ 2015 (Autodesk^®^ Corp., USA). 3D-structures for the production of negative molds were then created in three steps. First, the previously made 2D-layout was extruded to a desired channel depth. Second, the extruded part was boolean subtracted from a virtual object-slide shaped matrix, forming a negative mold. In the final step, the constructed 3D-structures (e.g., ramps) were separately added to a desired mold location and fused with the final model.

### B. Mold fabrication

The molds were fabricated on fused silica slides, 76 × 26 × 1 mm, with the FEMTOPRINT^®^ technology, which utilizes a femtosecond laser in the near-IR (*λ*) = 1030 nm) to tailor the properties of the glass substrate. A f100a Head machine (FEMTOprint SA, Switzerland) was used. The laser is focused with a 20 × (NA = 0.4) objective and prints the CAD designs of the microfluidic chips within the fused silica slides. Afterwards, the slides were immersed within a 2.5% HF solution in water for 8 h to remove the exposed material. Finally the chips were rinsed in de-ionized (DI) water and ultrasound to remove all debris and the structures were verified by light interferometry.

### C. PDMS chip fabrication

As the 3D-glass master is a negative mold (channels going in), an intermediate positive working mold was produced with PDMS (Sylgard 184, Dow Corning). For making master PDMS molds, a 5:1 mixture of base and curing agent was poured onto the glass mold, degassed in a vacuum desiccator, and then thermally polymerized at 70 °C for 3 h. The polymerized PDMS was peeled off from the mold and subsequently treated with Trichloro(1H,1H,2H,2H-perfluorooctyl)silane (Sigma).

PDMS at a standard 10:1 mixture of base and curing agent was poured onto the PDMS negative mold, degassed in a vacuum desiccator, and then thermally polymerized at 70 °C for three hours. The polymerized PDMS was peeled off from the mold, micropunched for fluidic connections, and plasma bonded (Zepto, Diener) to a microscope glass slide. The channels were treated with Novec 1720 (3M) for hydrophobization. The electrodes were made by flowing low melting solder (indium alloy no. 19, Indium Corp.) into the electrode microchannels. Optical fibers (FC fiber patch cable, multimode, 50 *μ*m core diameter, 0.22 NA, Thorlabs, and fiber patch cord, single mode, polarization maintaining, LASOS) were cleaved with a fiber scribe to obtain a flat surface at the fiber tip and stripped to obtain the core fiber plus cladding. The fibers were positioned manually into the chip through fiber guide channels. The air gap between the fiber tip and PDMS was removed by injecting uncured PDMS through specific inlets and letting it cure at room temperature overnight.

### D. Reagents

The aqueous phase consisted generally of fluorescent dyes (carboxyfluorescein, Sigma and DY-395XL, Dyomics) and/or microbeads (∅6 *μ*m, Distrilab) prepared and diluted in phosphate buffered saline (PBS, pH 7.4). For droplets with microorganisms, complex media was used for co-culturing of *Streptomyces* sp. and *E. coli*. Novec (HFE7500, 3M) oil with 0.5% Picosurf 1 (Dolomite) was used as a continuous phase for droplet generation.

### E. Microfluidic actuation and imaging

Fluids were actuated using both a syringe pump (neMESYS, Cetoni) and/or a pressure pump (OB-1, Fluigent), depending on the applications. Polytetrafluoroethylene (PTFE) tubing was used for fluidic connections. A high-speed camera (EoSens 4-CXP, Mikrotron) was used for imaging on an inverted microscope (Axio Observer Z1, Carl Zeiss) or a stereo microscope (Stemi 2000, Carl Zeiss). Fluorescence imaging was performed with a PCO.edge (PCO) camera and a Spectra-X (Lumencor) light source on the inverted microscope.

## IV. CONCLUSION

Here, we have presented a simple, yet powerful strategy to simplify, diversify, and make more broadly accessible the production of 3D-microfluidic chips. 3D-glass molds integrate the advantages of high resolution prototyping with the ease of production and replication offered by soft lithography. It also facilitates the manufacture of multilevel structures, even with the generation of ramps (gradients of confinement),^38,39^ which greatly improved key droplet microfluidic operations. This technique enables novel possibilities that can be exploited not only with droplets, but in many other applications of microfluidics^40^ (e.g., cell observation, cultivation^41,42^ and isolation,^14^ tissue and organs on a chip,^43^ and fluidic control and actuation^44^).

Due to the high sub-micron resolution, surface and material quality, this option provides an improved alternative for the fabrication of 3D-printed microfluidic structures in comparison to the prevalent state of the art.^15,16^ While we focused on PDMS-based soft-lithography, other materials can be used for replica molding. Furthermore, the 3D-glass structures present a negative structure, it is also possible to bond them to glass or a PDMS slab for direct usage as chips, thus allowing the utilization of structures that cannot be reproduced with replica molding (e.g., microstructures in the channels,^45,46^ or optical elements like waveguides, mirrors, filters, lenses, etc.).^47,48^ Given these possibilities, chip fabrication is no longer the bottleneck for the adoption of microfluidic approaches—specially by non-microfluidic labs—but rather an opportunity for the implementation of novel functionalities.

## V. SUPPLEMENTARY MATERIAL

See supplementary material for interferometry data, CAD designs, and videos of the presented chips and operations.
